# Epidemiology, determinants, and microbiological profile of implant-associated infections following intramedullary nailing in a tertiary hospital in Ethiopia

**DOI:** 10.1007/s00402-026-06535-6

**Published:** 2026-10-14

**Authors:** Abebaw Muhabaw Zegeye, Suleiman Ayalew Belay, Ephrem Bekele Kebede, Birlew Teshome Alemayehu, Sileshi Serebe Zeleke, Yohannis Derbew Molla

**Affiliations:** University of Gondar, Gonder, Ethiopia

**Keywords:** Intramedullary nailing, Implant-associated infection, Long bone fractures, Epidemiology, Determinants

## Abstract

**Background:**

Implant-associated infections after intramedullary nailing (IMN) are a major cause of morbidity in patients with long bone fractures. This study aimed to determine the magnitude, determinants, and microbiological profile of such infections in a tertiary hospital in Ethiopia.

**Methods:**

A hospital-based cross-sectional study was conducted among 260 patients who underwent IMN for lower extremity long bone fractures between January 2021 and December 2023. Clinical data, operative details, and microbiological results were collected, and logistic regression was applied to identify determinants of infection.

**Results:**

The overall infection rate was 8.46% (95% CI: 5.38–12.53; 22/260), comprising 4.62% deep and 3.85% superficial infections. We found that emergency surgery and hospital stay longer than 14 days were independently associated with an increased risk of infection. Among the limited number of culture-positive infections, Gram-negative bacteria predominated, with Escherichia coli as the most frequently identified pathogen.

**Conclusion:**

The infection rate was higher than in many other settings, underscoring the need for improved perioperative infection prevention and reduced hospital stays. Findings highlight the importance of perioperative optimization and hospital infection control in LMICs.

## Introduction

Lower extremity long bone fractures, particularly of the femur and tibia, are among the most common injuries encountered in orthopedic and trauma centers and represent a leading cause of hospital admission [1, 2]. These fractures also impose a substantial social and economic burden [3]. They frequently result from high-energy mechanisms such as road traffic accidents, falls from height, and ballistic trauma [1, 2, 4, 5].

Intramedullary nailing (IMN) is the preferred surgical technique for stabilizing these fractures because of its favorable biomechanical properties and relatively low complication rates [1, 6–10]. Nevertheless, implant-associated infections remain a serious postoperative complication, leading to increased morbidity, prolonged hospitalization, higher healthcare costs, and, in severe cases, revision surgery or limb loss [11–13].

Globally, studies on infection following IMN have generally focused on specific bones (femur or tibia) or fracture types (open versus closed). Reported infection rates vary widely. A multicenter prospective cohort study estimated an overall infection rate of 2.5% following tibial and femoral IMN surgeries [14]. Similarly, retrospective cohort studies have reported rates of approximately 6.7% after IMN for long bone fractures [9]. For open fractures, the risk is higher: a systematic review of 32 studies involving 3,060 patients reported infection rates of 5–16% for open tibial shaft fractures [15], while a meta-analysis of 17 studies on open femoral shaft fractures found rates of 5–6% [6]. In general, studies of fracture fixation surgeries have reported infection rates between 5% and 6% [3, 16].

In low- and middle-income countries (LMICs), a large multicenter study analyzing 46,113 IMN procedures reported relatively low crude infection rates of 0.8–1.5%, with femoral fractures less affected than tibial fractures [7]. However, these figures likely underestimate the true burden due to methodological limitations such as incomplete follow-up and underreporting, as many patients in resource-constrained settings do not return unless complications arise. Indeed, when only patients with documented follow-up were included, infection rates increased substantially—up to 6.9% for tibial fractures—suggesting that the actual burden in LMICs may be higher than crude estimates indicate [7].

In Ethiopia, evidence remains limited. The few studies available have reported infection rates ranging from 4% to 9% following IMN for lower extremity long bone fractures [17, 18]. However, these studies were restricted in scope and did not explore in detail the risk factors or microbiological characteristics of implant-associated infections.

Understanding the microbiological profiles and antimicrobial susceptibility patterns of pathogens responsible for implant-associated infections is critical for guiding empirical antibiotic therapy and informing infection prevention strategies. Furthermore, multiple risk factors—including high-grade open fractures (Gustilo-Anderson), delayed surgical intervention beyond 24 h, tobacco use, diabetes mellitus, prolonged operative time, and omission of prophylactic antibiotics—have consistently been associated with an increased risk of infection [7, 9, 14, 16]. Yet, little is known about the relative importance of these factors in the Ethiopian context.

To address these gaps, this study was conducted to determine the magnitude of implant-associated infections following IMN surgeries for long bones at a tertiary hospital in Ethiopia, to identify patient, injury, and perioperative factors associated with infection, and to describe the microbiological profile and antimicrobial resistance patterns of pathogens isolated from infected cases. By generating such evidence, this research seeks to support clinical decision-making, optimize perioperative care, and inform infection prevention strategies in resource-limited settings.

## Methods and materials

### Study setting and design

This hospital-based cross-sectional study was conducted at the University of Gondar Comprehensive Specialized Hospital (UOG CSH), a tertiary referral center located in Northwest Ethiopia.

### Study population

The study population included all patients who underwent IMN surgeries for fractures of the femur or tibia between January 2021 and December 2023.

### Eligibility criteria

Patients were eligible for inclusion if they received IMN surgery for tibial or femoral fractures at UOG CSH during the study period. Patients referred to UOG CSH after undergoing IMN surgery at other institutions, as well as those with pathological fractures, were excluded.

### Sample size and sampling technique

**Fig. 1:**
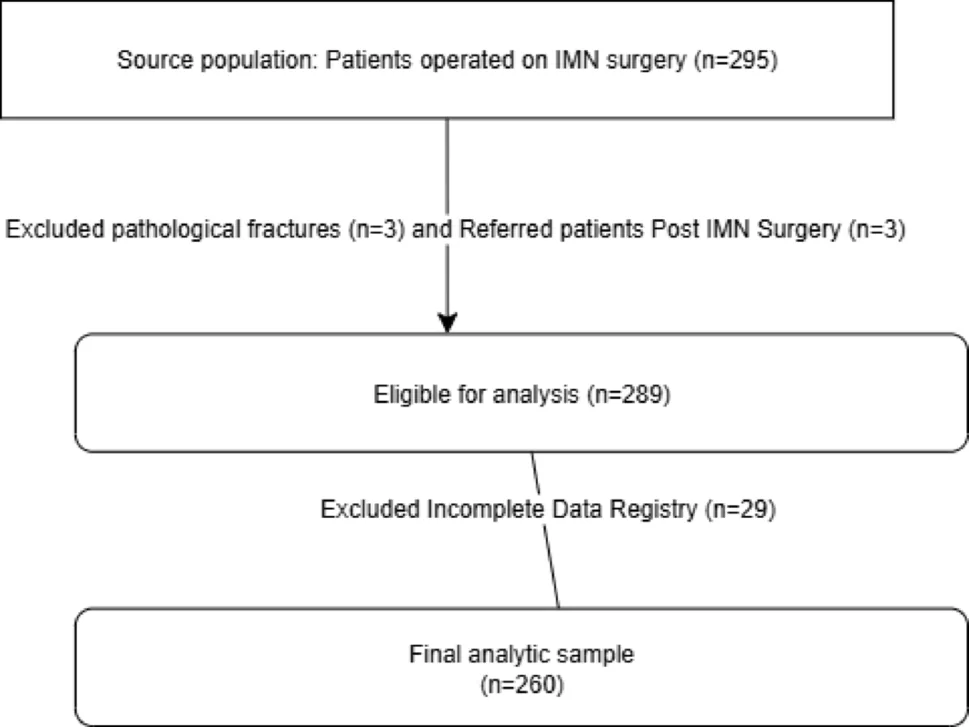
Flow diagram showing patient selection process

A total of 295 patients who underwent IMN surgery were initially identified. Of these, 6 patients were excluded: 3 due to pathological fractures and 3 who were referred from other hospitals after undergoing IMN surgery. This yielded 289 eligible cases. Subsequently, 29 patients were excluded due to incomplete data registry, resulting in a final analytic sample of 260 patients (Fig. 1).

### Study variables

The primary outcome variable was the occurrence of implant associated infection following IMN surgery, documented by the operating surgeon based on Metsemakers et al. criteria [19].

Independent variables included sociodemographic characteristics (age, sex, residence, and occupation), clinical presentation factors (systemic comorbidity status, initial hemoglobin level, and time from injury to hospital presentation), injury-related characteristics (mechanism of injury, polytrauma status, bone fractured, fracture type, and Gustilo-Anderson classification for open fractures), and perioperative management variables (timing of antibiotic initiation and initial debridement for open fractures, staging of IMN surgery, timing of definitive IMN surgery, type of IMN surgery, reduction technique for closed fractures, and duration of hospital stay).

### Perioperative antibiotic prophylaxis

Perioperative antibiotic prophylaxis was routinely administered to patients undergoing intramedullary nailing at the University of Gondar Comprehensive Specialized Hospital in accordance with the Ethiopian Standard Treatment Guideline for General Hospitals. For closed fractures requiring internal fixation, cefazolin 1–2 g was administered intravenously preoperatively, followed by doses every 8 h for two additional doses. For Gustilo–Anderson type I and II open fractures, cefazolin 1 g was administered intravenously preoperatively and continued every 8 h, with antibiotics discontinued 24 h after wound closure. For Gustilo–Anderson type III open fractures, cefazolin with additional gram-negative coverage (ceftriaxone plus gentamicin) was used, with antibiotics discontinued after 72 h or within 24 h after soft-tissue closure, whichever occurred first [20].

Because prophylactic antibiotic administration was routinely provided according to this standardized protocol, there was no meaningful between-patient variation in prophylaxis status that would permit its independent association with infection to be estimated; therefore, prophylaxis status was not included as an independent predictor in the regression analysis. For open fractures, the time from injury to initiation of antibiotic therapy was recorded separately because this timing varied between patients and was analyzed as a potential determinant of infection.

### Operational definitions

*Intramedullary nailing* was defined as a major orthopedic procedure involving theinsertion of a metallic rod into the medullary cavity of the femur or tibia, secured with proximal and distal interlocking screws.

*Implant-associated infection* was defined according to the Fracture-Related Infection (FRI) consensus criteria proposed by Metsemakers et al. [19]. Deep infection referred to infection involving the fracture site, implant, or bone, whereas superficial infection was limited to the skin and subcutaneous tissues without evidence of implant involvement. Since this was a retrospective chart review, all cases labeled as implant-associated infection in the medical records by the operating surgeon were accepted under the assumption that the attending surgeons applied these criteria.

*Emergency IMN surgery* For this study, emergency surgery was defined operationally as any IMN procedure documented as “emergency” in the operation note, regardless of the underlying clinical indication. This included cases performed within a short timeframe after hospital presentation due to urgent clinical need (e.g., open fracture, neurovascular compromise, compartment syndrome) as well as procedures expedited for institutional or logistical reasons (e.g., day-time availability of the operating theater, limited elective slots).

*Elective IMN surgery* Elective surgery was defined as any IMN procedure documented as “elective” in the operation note, generally performed after initial stabilization and scheduling, with sufficient time for preoperative evaluation and preparation.

### Data collection procedures and ethical clearance

Patient chart review was conducted from February 05, 2024, to May 30, 2024. Data were collected retrospectively from patient medical records using a pretested structured questionnaire. None of the authors had access to patient identifiers; all data were fully de-identified before analysis. Ethical clearance was obtained from the Institutional Review Board (IRB) of the UOG, and administrative permissions were secured before data collection.

### Data management and analysis

Following data collection, entries were coded and input into EpiData version 3.1, then exported to Stata version 17.0 for statistical analysis. Data cleaning procedures were performed to ensure accuracy, consistency, and completeness. Descriptive statistics were presented using frequencies, proportions, and graphical summaries.

Binary logistic regression was employed to identify factors associated with implant-associated infections. Candidate Predictor variables for multivariable logistic regression were selected based on bivariate analysis (*P* ≤ 0.2). Variables with sparse observations-such as Staged IMN surgeries, comorbidity status, smoking history, and closed reduction technique, were excluded due to insufficient observation (< 5% of total cases), which could compromise model stability. During multivariable logistic regression, certain categories such as bullet injury under the variable mechanism of injuries, were omitted due complete separation (perfect prediction), preventing stable estimation of regression coefficients, which prevented reliable estimation of adjusted odds ratios. These omissions were documented and interpreted accordingly in the Results section. Collinearity was assessed using variance inflation factors (VIFs) and linear regression diagnostics. The variable closed vs. open fracture was excluded due to its collinearity with Gustilo-Anderson grade of the open fractures, which was retained for its broader clinical relevance and stronger statistical performance. Gustilo-Anderson classification was analyzed only among patients with open fractures because the classification is not applicable to closed fractures. The variables retained in the final multiple logistic regression model included: age group, duration of injury at presentation, GA grade of the open fractures, time to initial debridement, type of IMN surgery (elective vs. emergency), duration of hospital stay, mechanism of injury, and poly trauma status (Table 3). The strength of association between independent variables and the outcome was assessed using odds ratios (OR) with 95% confidence intervals (CI), and statistical significance was set at *p* < 0.05. Patients were routinely followed for an average of 2 years through postoperative orthopedic clinic visits according to institutional practice. Infection outcomes were identified from both inpatient records and documented outpatient follow-up visits. Loss to follow-up was minimal (< 5%), and was not expected to materially influence estimation of infection occurrence.

## Results

### Socio-demographic and clinical characteristics

This study included a total of 260 patients who underwent intramedullary nailing (IMN) surgeries for tibial or femoral fractures between January 2021 and December 2023 at the UOG CSH. Age was categorized into three groups—young adults (18–30 years), middle-aged adults (31–60 years), and geriatric patients (≥ 61 years)—to facilitate subgroup comparisons and account for the non-normal distribution of age. Among these, nearly 60% were young adults aged 18–30 years, while only 17 patients (6.54%) belonged to the geriatric age group. A substantial proportion—196 patients (75.38%)—resided in rural areas. In terms of sex distribution, 217 patients (83.46%) were male, and 43 (16.54%) were female, reflecting the predominance of male trauma cases in the study setting. Regarding occupation, nearly half of the participants (124, or 47.69%) were farmers, followed by students, housewives, others, and merchants (Table 1).

**Fig. 2:**
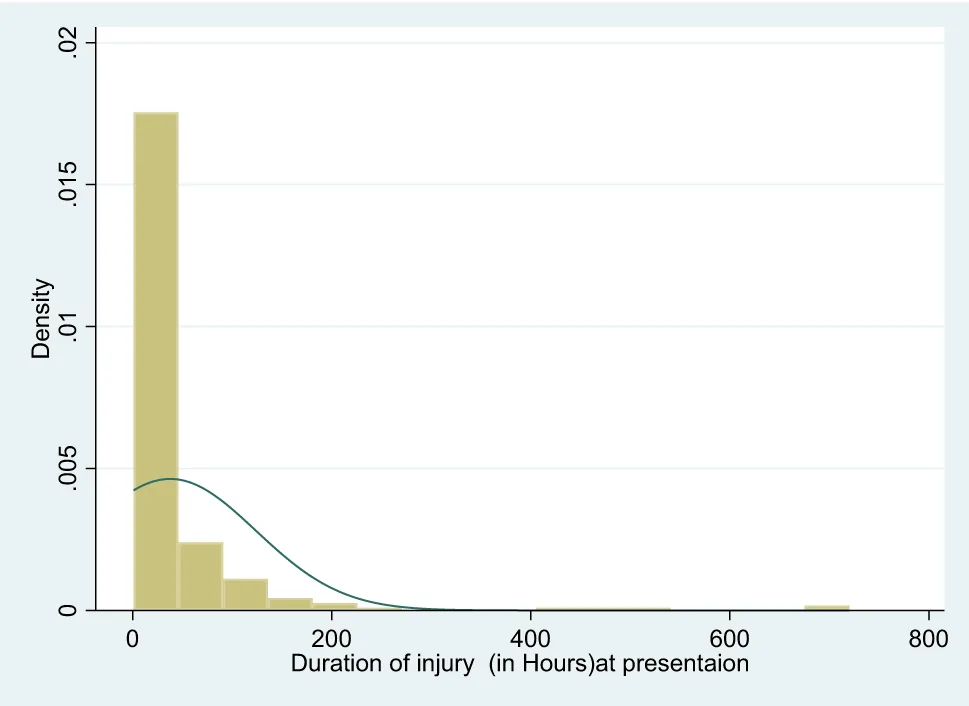
The histogram showed the distribution of duration of presentation following injury

**Fig. 3:**
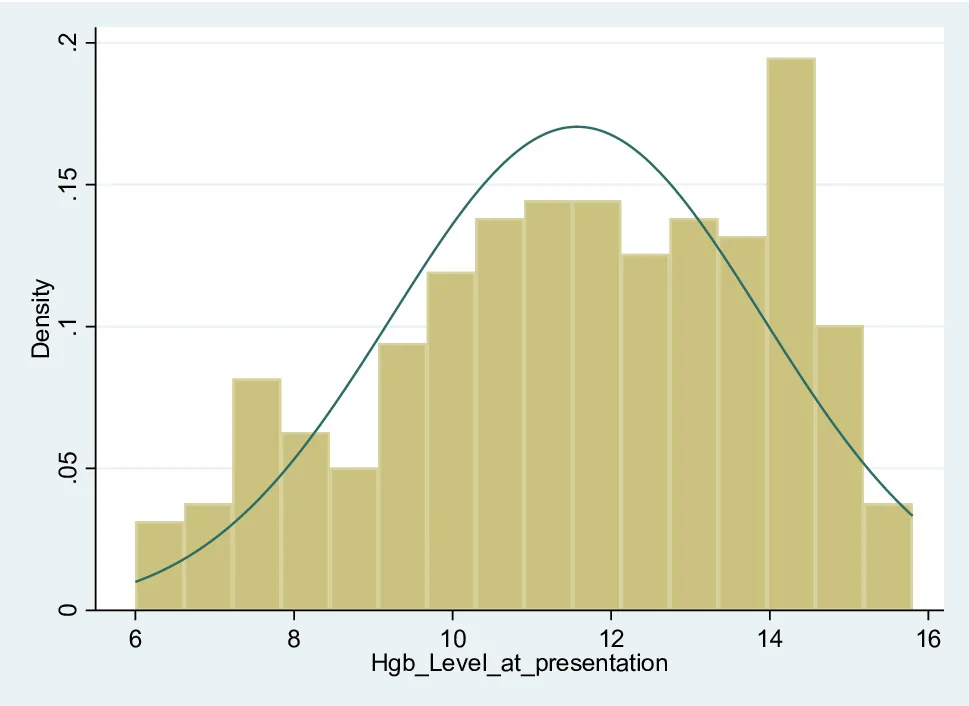
Initial Hemoglobin level (g/dl) at presentation

Clinical Characteristics of the study were organized thematically to enhance clarity and relevance. The domains included duration of injury presentation, hemodynamic status, injury profile, perioperative management, surgical timing, postoperative wound care, and hospital course. Variables were summarized using frequencies and percentages, with categorization applied to skewed distributions to support both analytic precision and clinical interpretation (Table 1). The median time from injury to hospital presentation was 12 h (IQR: 5.5 –24 h). The distribution was highly right-skewed (skewness = 5.53) (Fig. 2). Categorizing duration of injury presentation into three (within 12 h, 13–48 h, and after 48 h), 131(50.38%) of cases arrived at UOG CSH within 12 h of injury whereas 37 cases (14.23%) came after two days of injury. The hemoglobin level at presentation ranged from 6.0 to 15.8 g/dL, with a mean of 11.57 ± 2.34 g/dL and a median of 11.8 g/dL. Among the 260 participants, 64 patients (24.6%) had hemoglobin levels below 10 g/dL, suggesting a notable burden of anemia at the time of admission, which may influence infection risk and wound healing outcomes (Fig. 3).

Of the 260 patients who underwent IMN procedures in the tibia or femur, 165 (63.46%) had femoral fractures, while the remaining 95 (36.54%) sustained tibial fractures. A total of 165 cases (63.46%) were classified as open fractures. Among these, 132 patients (80%) presented with high-grade open fractures, corresponding to Gustilo-Anderson classifications IIIA, IIIB, or IIIC (Table 1).

**Fig. 4:**
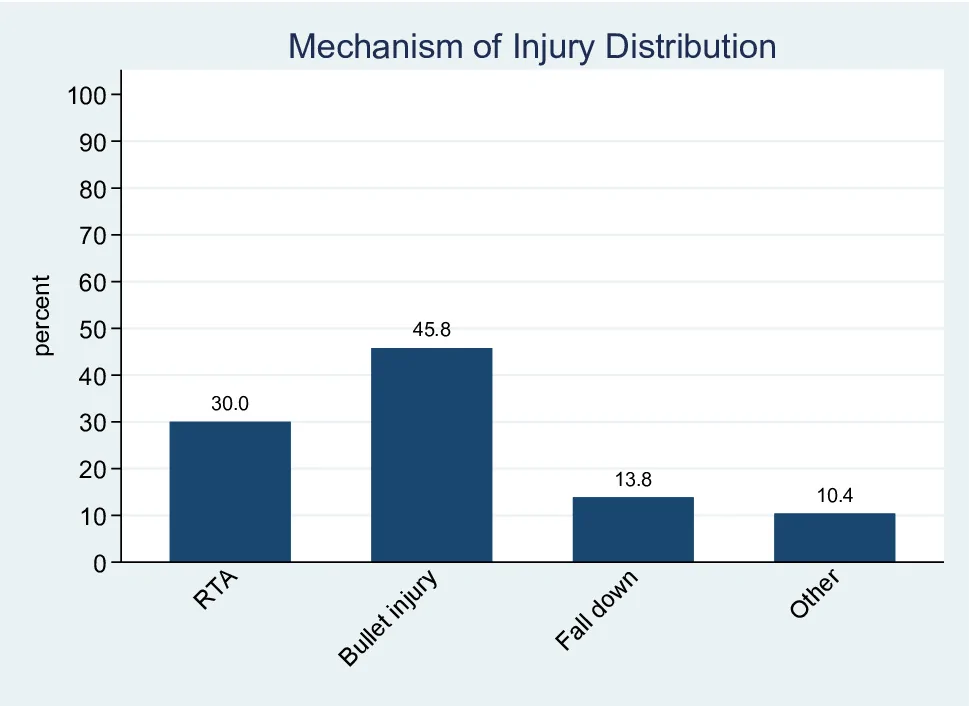
Percentage distribution of mechanism of injury among IMN patients

Of the 165 patients with open fractures, 72 (43.64%) received prophylactic antibiotics within 6 h of injury, while the remaining 93 (56.36%) received antibiotics after 6 h. In contrast, a large majority—111 patients (67.27%)—underwent initial surgical debridement more than 24 h after injury, indicating substantial delays in early wound management (Table 1). Of the 260 patients who underwent IMN surgery for tibial or femoral fractures, bullet injuries accounted for the largest proportion, affecting 119 individuals (45.77%). Road traffic accidents (RTAs) were the second most common mechanism, responsible for 78 cases (30.00%). Polytrauma was documented in 43 patients (16.54%), while the remaining cases involved isolated fractures of either the tibia or femur. These distributions are summarized in Table 1 and visually depicted in Fig. 4.

**Fig. 5:**
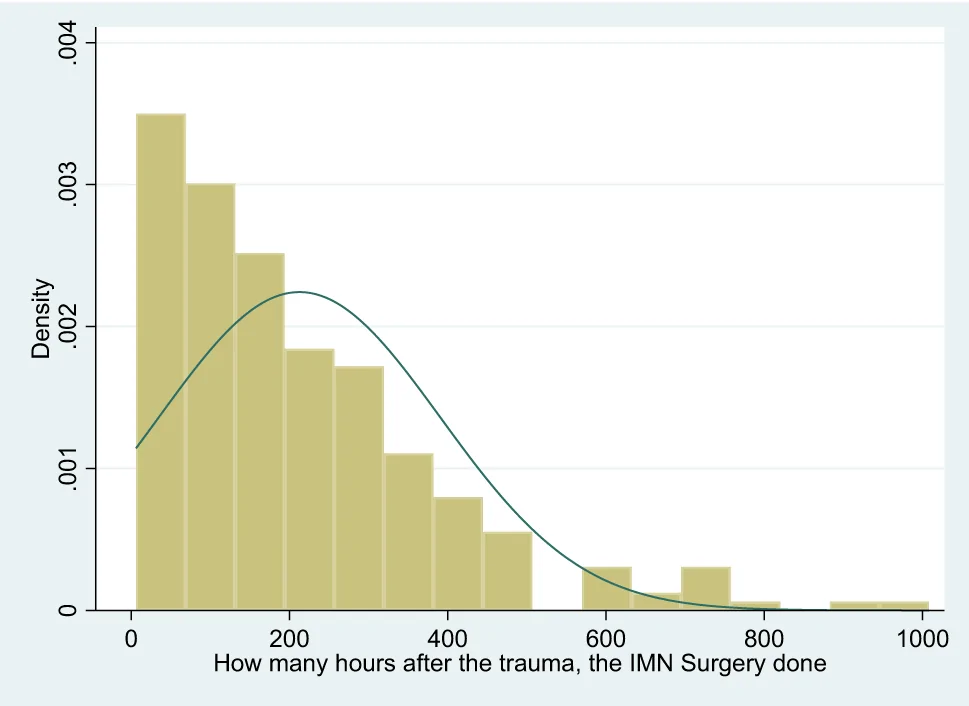
Timing of definitive IMN surgery in hours from trauma

The time to IMN surgery ranged from 6 to 1008 h, with a median of 180 h and a mean of 212.6 ± 177.9 h. The distribution was positively skewed, indicating that many patients experienced delays beyond the median (Fig. 5). For clinical interpretability, surgical timing was also categorized into early (≤ 48 h), delayed (49–168 h), and late (≥ 169 h) groups [21]. Over half of the patients (51.54%) underwent late surgery, while only 31.92% received early intervention (Table 1). In addition, 209 (80.38%) out of 260 IMN surgeries were elective, and the remaining 51 (19.62%) were operated on an emergency basis (Table 1). Among the 165 patients with open fractures, 84 (50.91%) underwent wound closure within 7 days of injury, while the remaining 81 (49.09%) had their wounds closed after 7 days (Table 1).

**Fig. 6:**
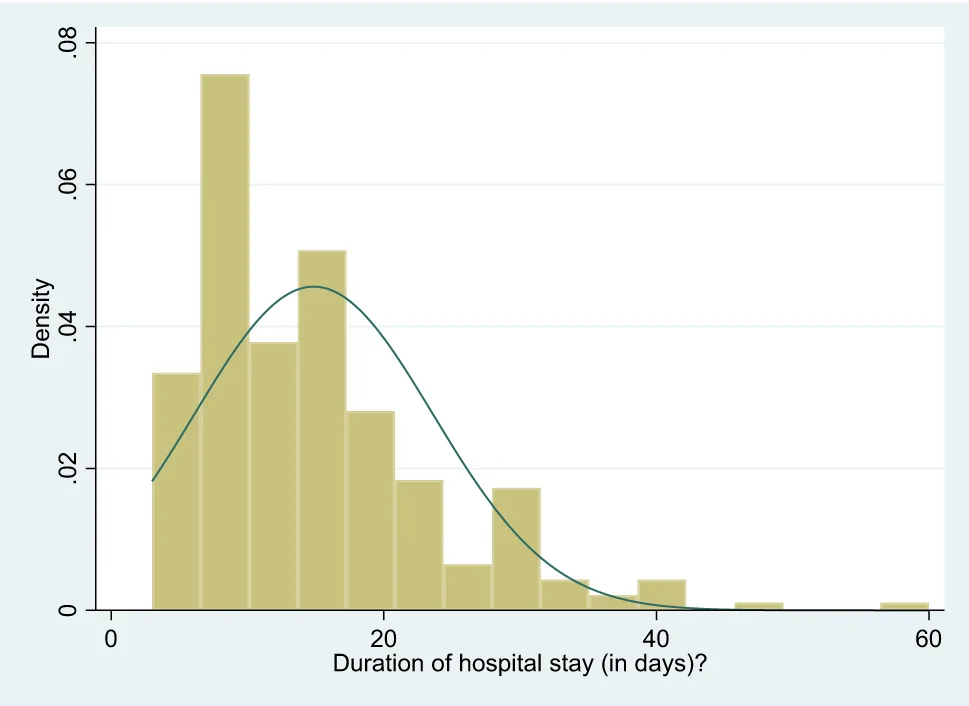
Duration of hospital stay in days

The duration of hospital stays among IMN-treated patients ranged from 3 to 60 days, with a median of 13 days and a mean of 14.85 days (SD ± 8.74). The interquartile range (IQR) was 9 to 19 days, indicating moderate variability (Fig. 6). Notably, 11.92% of patients were discharged within 7 days, while 47.31% stayed between 7 and 14 days, and 40.77% remained hospitalized for more than 14 days (Table 1).

**Table 1:** Demographic and clinical characteristics of study participants

Variable	Category	Frequency (*n* = 260)	Percent (%)
Age group (years)	18–30	153	58.85
	31–60	90	34.62
	> 61	17	6.54
Sex	Male	217	83.46
	Female	43	16.54
Residence	Rural	196	75.38
	Urban	64	24.62
Occupation	Farmer	124	47.69
	Merchant	21	8.08
	Student	43	16.54
	Housewife	38	14.62
	Other	34	13.08
Duration of injury at presentation (in hrs)	≥ 12	131	50.38
	13–48	92	35.38
	≥ 49	37	14.23
Initial Hemoglobin level (g/dl)	≤ 10	198	76.15
	< 10	62	23.85
Bone fractured	Tibia	95	36.54
	Femur	165	63.46
Nature of fracture	Closed	95	36.54
	Open	165	63.46
GA classification of open fractures (*n* = 165	Low (GA I and II)	33	20
	High (GA IIIA, IIIB, IIIC)	132	80
Time to initial debridement (in hrs) (*n* = 165)	≤ 24 h	54	32.73
	> 24 h	111	67.27
Time to antibiotic initiation for open fractures (in hrs) (*n* = 165)	≤ 6	72	43.64
	> 6	93	56.36
Mechanism of injury	Bullet	119	45.77
	RTA	78	30.00
	FDA	36	13.85
	Other	27	10.38
Poly trauma	Yes	43	16.54
	No	217	83.46
Time to IMN surgery (in hrs)	≤ 48	83	31.92
	49–168	43	16.54
	≥ 169	134	51.54
Type of surgery	Emergency	51	19.62
	Elective	209	80.38
Time to wound closure for open fractures (*n* = 165) (in days)	≥ 7	84	50.91
	> 7	81	49.09
Hospital stays (in days)	< 7	31	11.92
	7–14	123	47.31
	> 14	106	40.77

Bullet injury was the predominant cause, followed by road traffic accidents, falls, and other trauma. However, bullet injuries were omitted from multivariable regression because complete separation prevented reliable estimation of regression coefficients.

### Magnitude of implant-associated infections following IMN surgery

**Fig. 7:**
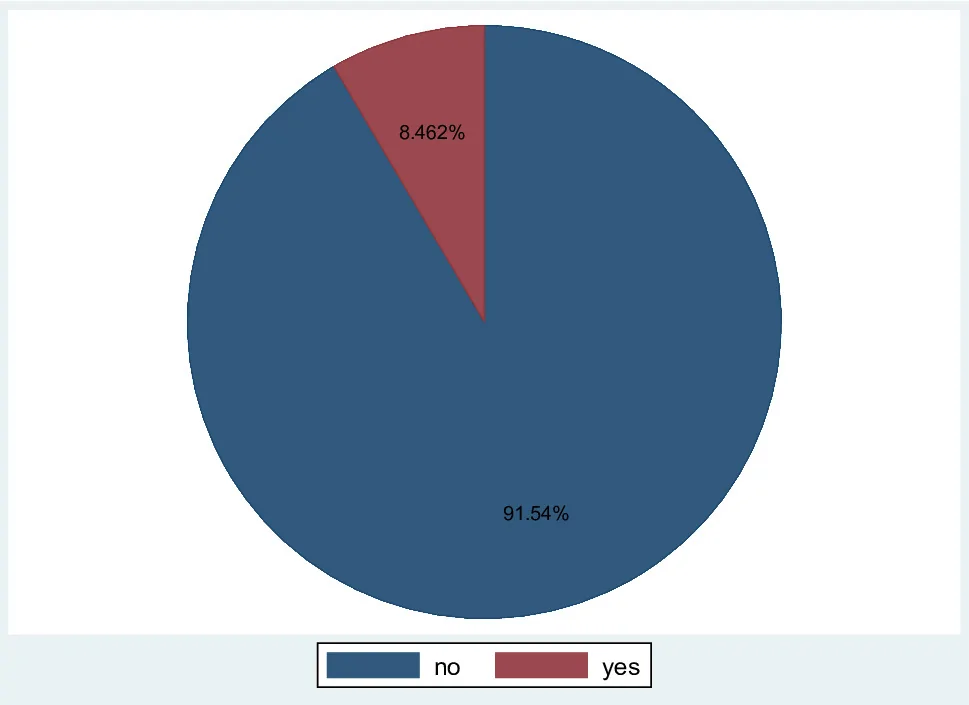
Proportion of IMN-associated infections

**Fig. 8:**
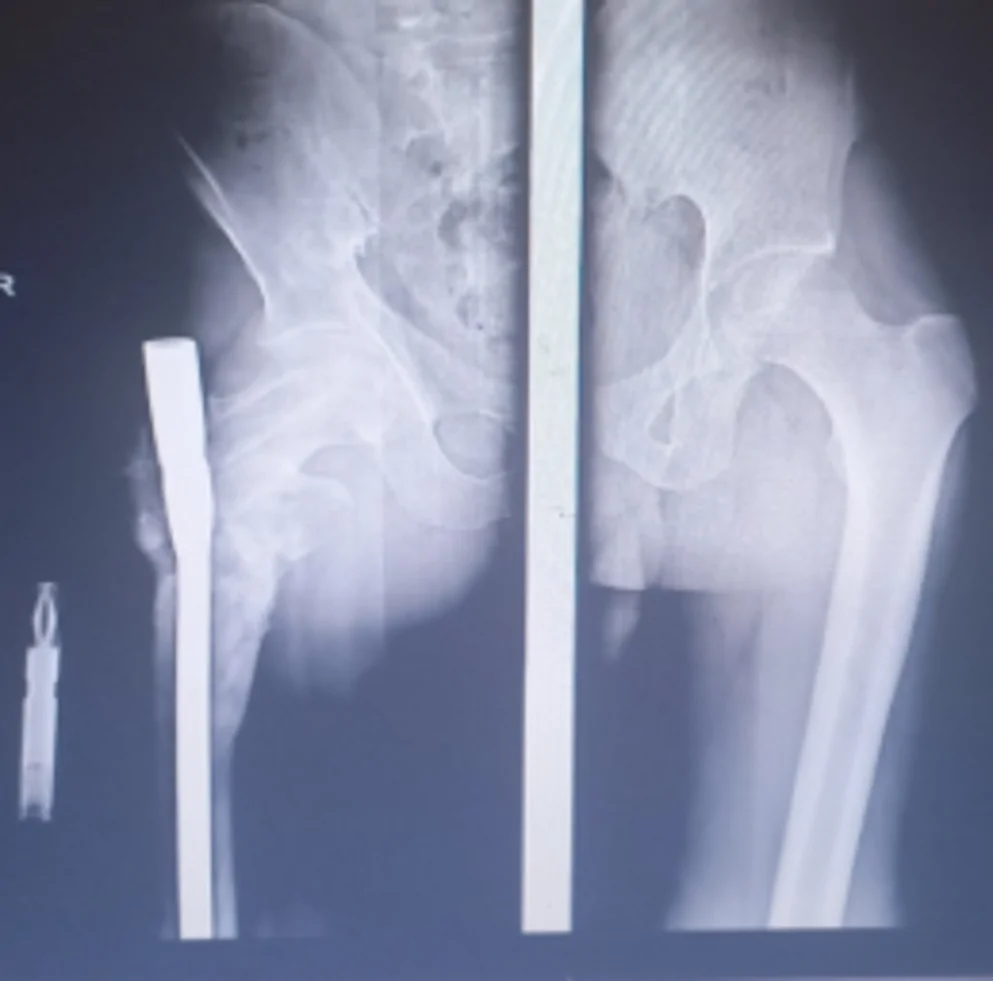
A 25 year old male patient with nail associated infection and loosening and fall of the locking screw

**Fig. 9:**
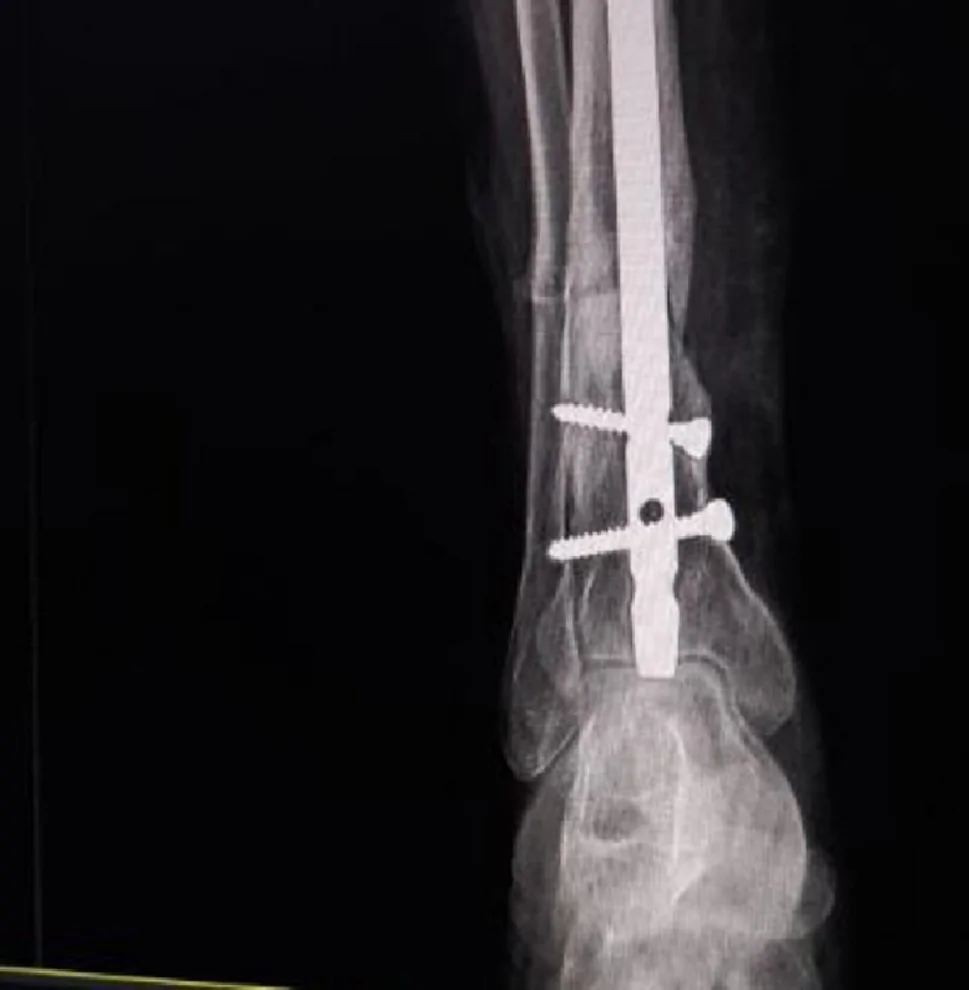
A 27 year old woman with nail associated infection after intramedullary nail was inserted for the diagnosis of tibiofibular fracture

Among 260 patients who underwent IMN surgery for tibial or femoral fractures, 22 cases of implant-associated infection were documented, yielding an overall infection rate of 8.46% (95% CI: 5.38%– 12.53%) (Fig. 7). Of these infections, 12 cases out of 260 (4.62%) were classified as deep infections, while the remaining 10 out of 260 (3.85%) were superficial (Figs. 8 and 9).

### Determinants of IMN-associated infections

The bivariate analysis identified several variables with potential associations to IMN-associated infections, using a liberal threshold of *p* ≤ 0.2 to capture clinically relevant trends. These variables included age category, duration of injury at presentation, nature of fracture, Gustilo-Anderson classification of open fractures, time to initial debridement, mechanism of injury, polytrauma status, type of IMN surgery, and duration of hospital stay (Table 2).

In the multivariate logistic regression model, two factors demonstrated statistically significant associations with IMN-associated infection. Patients who underwent emergency IMN surgery had eight times higher odds of developing infection compared to those treated electively (AOR = 8.075; 95% CI: 1.356–48.067; *p* = 0.022). Similarly, patients who remained hospitalized for more than 14 days had 27 times higher odds of infection compared to those discharged within 7 days (AOR = 27.542; 95% CI: 1.567–484.227; *p* = 0.023) (Table 3).

**Table 2:** Bivariate analysis of determinants of IMN-associated infection

Variable	Category	Infection Yes *n*	Infection No *n*	Crude OR (95% CI)	*P*-value
Sex	Male vs. female	18	199	0.88 (0.28–2.75)	0.828
Age	31–60 vs. 18–30	5	85	0.47 (0.17–1.32)	0.153
	≥ 61 vs. 18–30	0	17	Not estimable	–
Residence	Urban vs. Rural	5	59	0.89 (0.32–2.52)	0.830
Occupation	Overall variable	–	–	–	0.933
Duration of injury at presentation	13–48 h vs. ≤ 12 h	5	87	0.39 (0.14–1.09)	0.071
	≥ 49 h vs. ≤ 12 h	0	37	Not estimable	–
Initial hemoglobin	< 10 g/dL vs. ≥ 10 g/dL	7	55	1.55 (0.60–4.00)	0.362
Bone fractured	Tibia vs. Femur	7	88	0.80 (0.31–2.03)	0.631
Nature of fracture	Open vs. Closed	20	145	6.41 (1.46–28.08)	0.014
GA classification	Lower grade vs. Higher grade	1	32	0.19 (0.02–1.44)	0.107
Time to initial debridement	Early vs. Delayed	11	43	2.90 (1.12–7.50)	0.028
Time to antibiotic initiation	Early vs. Delayed	11	61	1.68 (0.66–4.31)	0.278
Mechanism of injury	Overall variable	–	–	–	0.076
Polytrauma	Yes vs. No	1	42	0.22 (0.03–1.70)	0.147
Type of IMN surgery	Emergency vs. Elective	9	42	3.23 (1.30–8.05)	0.012
Time to IMN surgery	49–168 h vs. ≤ 48 h	2	41	0.24 (0.05–1.11)	0.068
	> 168 h vs. ≤ 48 h	6	128	0.23 (0.08–0.63)	0.004
Time to wound closure	≤ 7 days vs. > 7 days	11	73	1.21 (0.47–3.08)	0.697
Hospital stay	Early vs. Delayed	–	–	0.49 (0.13–1.80)	0.281
	Intermediate vs. Delayed	–	–	0.42 (0.16–1.16)	0.096
	Overall variable	–	–	–	0.191

**Table 3:** Multivariate logistic regression analysis of determinants associated with implant-associated infections following IMN

Variable	Category	Infection status		Crude OR (95% CI)	P value
		Yes n	No n		
Sex	Male	18	199	0.09 (0.056–0.146)	0.829
	Female	4	39	0.10 (0.037–0.287)	
Age (years)	18–30	17	136	0.125 (0.076–0.206)	**0.048**
	31–60	5	85	0.059 (0.024–0.144)	
	≥ 61	0	17		
Residence	Rural	17	179	0.095 (0.058–0.156)	0.830
	Urban	5	59	0.085 (0.034–0.211)	
Occupation	Farmer	10	114	0.088 (0.046–0.167)	0.620
	Merchant	1	20	0.050 (0.006–0.373)	
	Student	3	40	0.075 (0.023–0.242)	
	Housewife	3	29	0.103 (0.031–0.340)	
	Other	4	33	0.121 (0.043–0.342)	
Duration of injury at presentation (in hrs)	≤ 12	17	114	0.149 (0.089–0.248)	**0.005**
	13–48	5	87	0.057 (0.023–0.142)	
	≥ 49	0	37	0.000	
Initial hemoglobin level (g/dl)	≥ 10	15	183	0.082 (0.048–0.139)	0.360
	< 10	7	55	0.127 (0.058–0.279)	
Type of bone fractured	Femur	15	150	0.100 (0.059–0.170)	0.631
	Tibia	7	88	0.080 (0.037–0.172)	
Nature of the fracture	Open	20	145	0.138 (0.086–0.220)	**0.005**
	Closed	2	93	0.021 (0.005–0.087)	
GA Classification	Lower class (Type I and Type II)	1	32	0.31 (0.004–0.229)	0.075
	Higher class (GA IIIA, B, C)	19		0.168 (0.103–0.273)	
Time to initial debridement (in hrs)	≤ 24	11	43	0.256 (0.131–0.496)	**0.024**
	> 24	9	102	0.088 (0.044–0.174)	
Time to antibiotic initiation for open fractures (in hrs)	≤ 6	11	61	0.180 (0.095–0.343)	0.276
	> 6	9	84	0.107 (0.054–0.213	
Mechanism of injury	RTA	5	73	0.068 (0.028–0.169)	0.147
	Bullet injury	17	102	0.167 (0.100-0.279)	
	Fall down injury	0	36	0.000	
	other	0	27	0.000	
polytrauma	Yes	1	42	0.107 (0.068–0.168)	0.1
	No	21	196	0.024 (0.003–0.173)	
Type of IMN surgery	Emergency	9	42	0.214 (0.104–0.440)	**0.008**
	Elective	13	196	0.066 (0.038–0.116)	
Time to IMN surgery (in hrs)	≤ 48	14	69	0.202 (0.114–0.360)	**0.002**
	49–168	2	41	0.049 (0.012–0.202)	
	> 168	6	128	0.047 (0.021–0.106)	
Time to wound closure for open fractures (in days)	≤ 7	11	73	0.150 (0.080–0.284)	0.697
	> 7	9	72	0.125 (0.063–0.250)	
Duration of Hospital stays (in days)	< 7	1	30	0.33 (0.005–0.244)	0.059
	7–14	8	115	0.100 (0.034–0.142)	
	> 14	13	93	0.140 (0.078–0.250)	

**Table 4:** Microbiological profile and antimicrobial resistance patterns of pathogens isolated from IMN-associated infections (*n* = 22)

Variable	Category	AOR (95% CI)	*P*-value
Age (years)	18–30	1	0.941
	31–60	1.05 (0.295–3.722)	
	≥ 61	1	
Duration of injury at presentation(hrs)	≤ 12	1	0.246
	13–48	0.431 (0.104–1.782)	
	≥ 49		
GA Classification	Lower class (Type I and Type II)	1	0.443
	Higher class (GA IIIA, B, C)	2.63 (0.221–31.377)	
Time to initial debridement (in hrs	≤ 24		0.318
	> 24	0.419(0.076–2.361)	
Mechanism of injury	RTA	1.047(0.181–6.045)	0.959
	Bullet injury	–	
	Fall down injury	1	
	other	–	
Polytrauma	Yes	0.230(0.022–0.245)	0.224
	No	1	
Type of IMN Surgery	Emergency	8.075(1.356–48.067)	**0.022**
	Elective	1	
Time to IMN surgery (in hrs)	≤ 48	1	
	49–168	1.002 (0.115–8.715)	0.998
	> 168	1.710 (0.236–12.396)	0.596
Duration of Hospital stays (in days)	< 7	1	
	7–14	2.620 (0.238–28.815)	0.431
	> 14	27.542(1.567-484.227)	**0.023**

Variable	Category	Frequency (*n*)	Percentage (%)
Culture & sensitivity performed	No	13	59.1%
	Yes	9	40.9%
Organisms isolated (*n* = 9, culture performed)	No growth	5	55.6%
	Growth	4	44.4%
Types of isolated organisms (*n* = 4 positive cultures)	Escherichia coli	2	50%
	Klebsiella pneumoniae	1	25.0%
	Non-lactose-fermenting Gram-negative rod	1	25. 0%
Antimicrobial Sensitivity (*n* = 4 positive cultures)	Meropenem, Ciprofloxacin	1	25. 0%
	Chloramphenicol	1	25.0%
	Gentamicin	1	25.0%
	Imipenem, ceftazidime	1	25.0%
Antimicrobial resistance (*n* = 4 positive cultures)	Ceftriaxone, ampicillin, ceftazidime	1	25.0%
	Azithromycin, Vancomycin	1	25.0%
	Cefuroxime, Cotrimoxazole, Ciprofloxacin, Tazobactam/Piperacillin	1	25.0%
	Gentamicin, Ceftriaxone, Ceftazidime	1	25.0%

Out of 22 infected cases, culture and sensitivity testing were performed in 9 (40.9%). Of these, 4 (44.4%) yielded growth. The most common pathogen was E. coli (50%), followed by K. pneumoniae (25%) and non-lactose fermenting Gram-negative rods (25%). The isolates showed sensitivity to meropenem, ciprofloxacin, chloramphenicol, gentamicin, imipenem, and ceftazidime, while resistance was observed against ceftriaxone, ampicillin, azithromycin, vancomycin, cefuroxime, cotrimoxazole, ciprofloxacin, and piperacillin-tazobactam (Table 4). Because only four patients yielded positive cultures, these microbiological findings should be interpreted cautiously and may not represent the complete microbiological spectrum of implant-associated infections at our institution.

## Discussion

This study found an overall implant-associated infection rate of 8.46% (95% CI: 5.38%–12.53%) following IMN surgery in the tibia or femur, consisting of 4.62% (*n* = 12) deep infections and 3.85% of superficial infections (*n* = 10).

Compared with Beza et al. [17], who reported a 4.18% infection rate after long-bone IMN, our observed rate is substantially higher. This discrepancy likely reflects the greater prevalence of open fractures in our cohort (approximately 64% [165/260] versus 24% in Beza et al.

A retrospective cross-sectional study at Saint Paul’s Hospital Millennium Medical College in Addis Ababa similarly reported an overall infection rate of 9.3% [18], which aligns closely with our findings. However, the prevalence of deep infections in our series (4.62%) exceeded that reported by Desta et al. (3.45%) (Desta et al. 2023). This difference may stem from fracture severity: GA III A injuries predominated in our study, whereas GA II fractures were most common in Saint Paul’s cohort.

By contrast, Young et al. [7] documented a markedly lower overall IMN infection rate of 1%. Their study’s reliance on an incomplete follow-up registry (Sign Online Surgical Database) may have underestimated true infection rates. Similarly low rates have been observed in high-resource settings [22–24], reflecting differences in perioperative environments, operating room sterility protocols, and patient baseline characteristics. Moreover, those studies did not focus on specific bone or implant types, limiting direct comparability.

Conversely, several reports describe higher infection rates after IMN surgery to long bones of lower extremity than ours—11.9% in Whiting et al. and 12.9% in Adesina et al. [25, 26]. These elevated rates are attributable to study populations confined to open tibial shaft fractures [26] or combined open femoral and tibial shaft fractures [25], both of which carry greater contamination risk and soft-tissue compromise.

Our multivariate analysis identified emergency surgery, and a hospital stay longer than two weeks as significant predictors of infection following intramedullary nailing of femoral and tibial fractures (Table 3). The association between prolonged hospitalization and increased risk of infection is consistent with the findings of Weaver et al. [27], who reported that a hospital stay of more than 10 days was statistically significant in predicting orthopedic surgical site infections. Similarly, a prospective study of the bacteriological profile and risk factors of infection after internal fixation of closed fractures of long bones identified that hospitalization for more than two weeks is an independent risk factor for the development of infection [28]. Prolonged hospitalization may increase infection risk through extended exposure to hospital pathogens, repeated invasive procedures, and greater vulnerability related to comorbidities.

Similarly, our finding that emergency surgery is an independent risk factor for infection aligns with the results of Rezae et al. [29], who demonstrated that emergency IMN for femoral and tibial shaft fractures was associated with a higher risk of postoperative infection. This may be due to higher wound contamination risk and limited preoperative preparation in emergency surgeries.

However, the role of emergency surgery as a determinant of infection remains controversial. In contrast to our findings, studies conducted by Ercole et al. and Maksimovic et al. did not identify emergency surgery as an independent predictor of infection in orthopedic operations [30, 31]. These discrepancies may reflect differences in study populations, case selection, perioperative protocols, and infection control measures across institutions.

Among the limited number of culture-positive infections, all isolates were Gram-negative organisms, predominantly Escherichia coli, Klebsiella pneumoniae, and non-lactose-fermenting gram-negative rods (Table 4). This finding contrasts with the results of other studies, where Staphylococcus species were reported as the most frequently isolated pathogens, followed by gram-negative organisms [28, 31, 32]. Several factors may explain this discrepancy. First, in many cases, cultures were obtained after the initiation of antibiotic therapy, which may have suppressed the growth of gram-positive bacteria. Second, the limited availability of culture media in our setting may have restricted the growth of a wider range of organisms. Finally, contamination during the collection or processing of specimens could have influenced the spectrum of organisms identified. These findings highlight the need for timely culture sampling, improved microbiological facilities, and strict adherence to aseptic techniques, as they are essential for guiding appropriate antibiotic selection and improving infection management in our hospital. Although prolonged hospitalization was independently associated with infection, reverse causality cannot be excluded because infection itself may prolong hospital stay. Therefore, this association should not be interpreted as causal in the context of our retrospective cross-sectional design.

Perioperative antibiotic prophylaxis was routinely administered according to the institutional protocol based on the Ethiopian Standard Treatment Guideline; therefore, prophylaxis status itself was not included as an independent predictor because there was no meaningful between-patient variation in its administration. Nevertheless, the retrospective nature of the study limited our ability to fully capture differences in the precise timing, duration, and adherence to prophylaxis, which may represent sources of residual confounding. Recent prospective evidence suggests that the generation of cephalosporin used for perioperative prophylaxis may be relevant to SSI risk in open fractures, with higher-generation cephalosporins not demonstrating additional protective benefit over first-generation agents [33]. Similarly, prolonged postoperative surgical antibiotic prophylaxis has not been shown to provide additional protection against SSI in orthopaedic surgery [34]. These findings provide relevant context for interpreting our results, although they were derived from different populations and should not be considered direct evidence from our cohort.

### Limitations

This study has several limitations that should be acknowledged. First, its retrospective cross-sectional design relied on secondary data from medical records, which may have been incomplete or inconsistently documented. This could have led to missing information on relevant variables such as nutritional status, glycemic control, and smoking history, which are known to influence infection risk. Second, culture and sensitivity testing were performed in less than half of the infected cases, limiting the ability to fully characterize the microbiological spectrum and antimicrobial resistance patterns. Third, although follow-up was generally adequate, variations in follow-up duration inherent to retrospective record review may have resulted in under-detection of late fracture-related infections. Fourth, only 22 infection events occurred, limiting statistical power and resulting in wide confidence intervals for some adjusted odds ratios; therefore, these estimates should be interpreted cautiously. Fifth, although only four positive cultures were obtained, this severely limits conclusions regarding the full microbiological spectrum of infections at our institution. Sixth, reverse causality cannot be excluded for the hospital stay variable, as infection itself may prolong hospitalization. Finally, as the study was conducted in a single tertiary hospital, the findings may not be generalizable to other institutions with different patient populations, perioperative protocols, or resource settings.

## Conclusion

This study demonstrated that the overall infection rate following intramedullary nailing of femoral and tibial fractures at UOG CSH was 8.46%, which is higher than rates reported in some high-resource settings but comparable to findings from other Ethiopian and regional studies. Multivariate analysis identified emergency surgery and prolonged hospital stay (> 14 days) as independent predictors of infection. although the culture yield was low, among the limited number of culture-positive infections, Gram-negative organisms predominated, with Escherichia coli and Klebsiella pneumoniae most commonly identified. This contrasts with the staphylococcal predominance reported in other settings, though the small number of positive cultures warrants caution in interpretation. These findings underscore the need for timely culture sampling, optimization of perioperative protocols, and strategies to minimize unnecessary hospital stays to reduce infection risk.

### Future directions

Future studies should adopt prospective cohort or longitudinal designs to allow more robust assessment of causality and better capture of late-onset infections. Expanding microbiological capacity, including the use of diverse culture media and molecular diagnostic tools, would help to more accurately define the spectrum of causative pathogens and their resistance patterns. Multicenter studies across Ethiopia and similar low-resource settings are also warranted to enhance generalizability and identify institution-specific infection control gaps. Additionally, intervention-focused research aimed at reducing delays to surgery, improving early wound management, and strengthening antibiotic stewardship will be critical for lowering the burden of implant-associated infections in this context.

## Data Availability

The datasets used and/or analyzed during the current study are available from the corresponding author on reasonable request.

## Abbreviations

AOR: Adjusted odds ratio
CDC: Centers for disease control and prevention
CI: Confidence interval
CSH: Comprehensive specialized hospital
FDA: Fall down accident
g/dl: gram per deciliter
GA: Gustilo -Anderson
Hrs: hours
IMN: Intramedullary nailing
IQR: Interquartile range
IRB: Institutional review board
LMICs: Low- and middle-income countries
N: number
OR: odds ratio
RTA: Road traffic accident
SD: Standard deviation
UOG: University of Gondar
VIF: Variance inflation factor
